# Successful Asymmetric Nasal High-Flow Therapy in CO₂ Narcosis Triggered by Pneumonia in an Elderly Patient: A Case Report

**DOI:** 10.7759/cureus.86401

**Published:** 2025-06-19

**Authors:** Keita Takahashi, Shigeto Ishikawa, Akari Kusaka, Hiroyuki Takeuchi, Tomohiko Akahoshi

**Affiliations:** 1 Department of Emergency Medicine, Fukuoka Kinen Hospital, Fukuoka, JPN; 2 Emergency and Critical Care Center, Kyushu University Hospital, Fukuoka, JPN

**Keywords:** acute co2 narcosis, asymmetric nhf, elderly patient care, high-flow nasal therapy, hypercapnia, type ii respiratory failure

## Abstract

The number of elderly people transported to the emergency department with respiratory failure is increasing. Some do not wish to receive invasive interventions, but when patients are unconscious, determining their treatment preferences can be challenging. Asymmetric nasal high-flow (NHF) therapy is a non-invasive respiratory support method that has shown effectiveness in type II respiratory failure, although there are no prior reports on its use for carbon dioxide (CO₂) narcosis. We report a case in which asymmetric NHF therapy was effective for CO₂ narcosis triggered by pneumonia in an elderly patient. A 96-year-old man with impaired consciousness was brought to the emergency department. Arterial blood gas analysis revealed respiratory acidosis, and imaging confirmed the presence of pneumonia and CO₂ narcosis secondary to pneumonia. Given the patient's advanced age, the goal was to avoid invasive intubation. Non-invasive ventilation (NIV) was considered, but deemed unsuitable because of excessive sputum and impaired consciousness. Therefore, asymmetric NHF therapy was initiated. The patient became responsive within 40 minutes, and his respiratory acidosis improved. After regaining consciousness, the treatment plan was discussed with the patient, and antibiotic therapy and asymmetric NHF were continued. For elderly patients, patient preference should be taken into account when making airway management decisions. Although NIV is commonly used for CO₂ narcosis, it is not suitable for certain patients, such as patients with excessive sputum and impaired consciousness. Asymmetric NHF is a noninvasive option that may help reduce the partial pressure of arterial CO_2_ and improve consciousness. This case suggests that asymmetric NHF may be a valuable therapeutic option for CO₂ narcosis in the elderly.

## Introduction

As populations in developed countries continue to age, the number of elderly patients presenting with respiratory failure is expected to increase [1]. Although positive pressure ventilation via mechanical ventilation is a well-established treatment for respiratory failure, it is associated with high mortality in elderly patients [2] and may lead to prolonged life support, which might not align with patient preferences. In such cases, non-invasive respiratory therapies may offer more appropriate and patient-centered options.

Asymmetric nasal high-flow (NHF) therapy is a non-invasive respiratory support technique that uses an asymmetric nasal cannula. Compared to standard NHF therapy, it is believed to more effectively flush carbon dioxide (CO₂) from the anatomical dead space [3]. Type II respiratory failure accompanied by CO₂ narcosis has a high one-year mortality rate of 45-50% and fatal complications in 15-25% [4], although mechanical ventilation and non-invasive ventilation (NIV) remain the standard treatments [5]. The efficacy of asymmetric NHF in this setting has not been established.

We report a case of an elderly patient with CO₂ narcosis secondary to pneumonia who was transported to the emergency services. In the present case, both mechanical ventilation and NIV were considered inappropriate because of the patient’s clinical condition. Asymmetric NHF therapy was initiated, which successfully improved CO₂ narcosis, allowing treatment to proceed in accordance with the patient’s wishes. This case suggests that asymmetric NHF therapy may be a useful alternative for the respiratory management of CO₂ narcosis in elderly patients.

## Case presentation

A 96-year-old man with a history of chronic heart failure, chronic kidney disease, and hypertension collapsed at a supermarket and was transported to the emergency department by an ambulance. Upon initial contact with the emergency medical services, his level of consciousness was Glasgow Coma Scale (GCS) E1V1M1, and oxygen saturation (SpO₂) was unmeasurable. Upon arrival at our hospital, the patient remained unresponsive (GCS E1V1M1). His vital signs were as follows: blood pressure of 152/84 mmHg, heart rate of 103 beats/minute, respiratory rate of 18 breaths/minute, and body temperature of 36.5°C; SpO₂ was still undetectable despite oxygen administration via a reservoir mask at 10 L/minute. His pupils were 2.5 mm bilaterally and reactive to light. On physical examination, coarse crackles were auscultated bilaterally, and sputum accumulation was noted in the oral cavity. Arterial blood gas analysis revealed a pH of 7.14, partial pressure of arterial carbon dioxide (PaCO_2_) of 74 mmHg, and bicarbonate (HCO_3_) of 25.2 mmol/L, indicating respiratory acidosis (Table 1).

**Table 1:** An arterial blood gas analysis on arrival at the emergency department (under reservoir mask oxygen at 10 L/minute). Respiratory acidosis due to hypercapnia was observed.

Parameters	Patient Value	Reference Ranges
pH	7.14	7.35–7.45
PaCO₂ (mmHg)	74	35–45
PaO₂ (mmHg)	229	80–100
Lactate (mmol/L)	3.4	0.5–1.6
Bicarbonate (mmol/L)	25.2	22–26
Base Excess (mmol/L)	-5.4	-2.0–2.0
Glucose (mg/dL)	147	73–109

**Figure 1:**
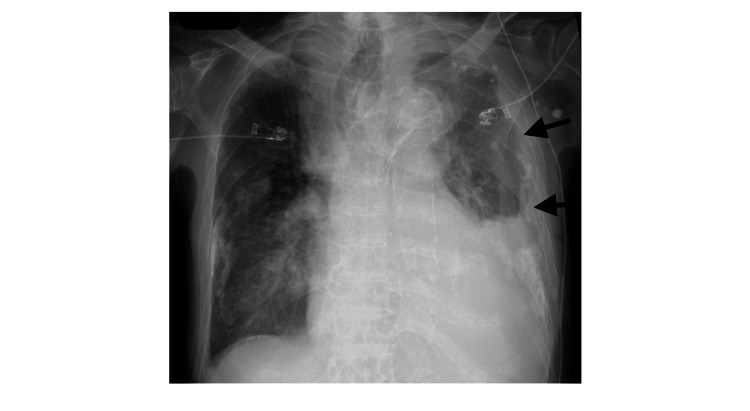
Chest radiography on arrival at the emergency department showing decreased translucency, predominantly in the left lung field (black arrows).

**Figure 2:**
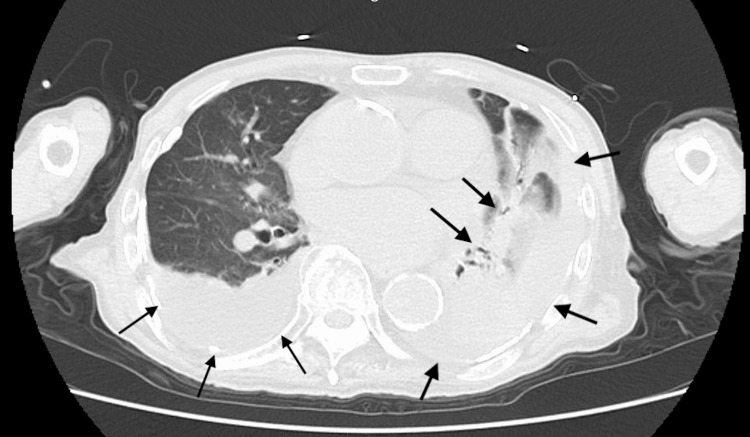
Chest CT images on arrival at the emergency department showing bilateral pleural effusions and infiltrative shadows predominantly in the left lung (black arrows).

Blood tests revealed an elevated white blood cell count (WBC) of 13,250/μL, C-reactive protein (CRP) of 10.14 mg/dL, and N-terminal pro-B-type natriuretic peptide (NT-proBNP) of 5,413 pg/mL, indicating an inflammatory response and possible heart failure. Echocardiography showed a reduced ejection fraction of 42% and an inferior vena cava (IVC) diameter of 17 mm without respiratory variation, further supporting the diagnosis of heart failure. Chest radiography revealed decreased translucency, predominantly in the left lung field (Figure 1). Head computed tomography (CT) was unremarkable, whereas chest CT revealed pulmonary infiltration and pleural effusion (Figure 2).

Based on these findings, the patient was diagnosed with CO₂ narcosis secondary to pneumonia and heart failure. Although he was found to possess personal items suggesting a family presence, no contact information was initially available. In such cases, our hospital makes a comprehensive policy decision based on a multidisciplinary approach. Based on the multidisciplinary consultation, considering his advanced age, we initially opted for non-invasive treatment while attempting to reach his family. The patient was unresponsive and had significant oral sputum retention, raising concerns about airway obstruction with NIV. In addition, his facial features (e.g., sunken cheeks) made mask fitting difficult. Therefore, we initiated respiratory support with the Optiflow® Duet system (Fisher & Paykel Healthcare Corporation Limited, Auckland, New Zealand) using asymmetric NHF therapy at a fixed flow rate of 50 L/minute and an initial fraction of inspired oxygen (FiO_2_) of 60%, along with intravenous antibiotics. Once SpO₂ became measurable, FiO₂ was gradually titrated to maintain the target SpO₂ between 94% and 98% while keeping the flow rate constant.

Forty minutes after initiating asymmetric NHF therapy (flow: 50 L/minute, FiO₂: 30%), the patient’s consciousness markedly improved to GCS E4V5M6. At that time, an arterial blood gas analysis showed a pH of 7.38, PaCO_2_ of 52 mmHg, and HCO_3_ of 30.8 mmol/L, indicating the resolution of respiratory acidosis (Table 2).

**Table 2:** Results of arterial blood gas analysis 40 minutes after the initiation of asymmetric nasal high-flow therapy (flow: 50 L/minute, FiO₂: 30%). Hypercapnia was reduced, respiratory acidosis improved, and the level of consciousness was restored.

Parameters	Patient Values	Reference Ranges
pH	7.38	7.35–7.45
PaCO₂ (mmHg)	52	35–45
PaO₂ (mmHg)	79	80–100
Lactate (mmol/L)	0.8	0.5–1.6
Bicarbonate (mmol/L)	30.8	22–26
Base Excess (mmol/L)	4.6	-2.0–2.0
Glucose (mg/dL)	125	73–109

The clinical course was consistent with CO₂ narcosis. Once the patient was alert and communicative, we confirmed that he did not wish to undergo invasive treatment. His family was successfully contacted. He resumed oral intake on hospital day 2. Although the pneumonia treatment was continued with antibiotics, his heart failure worsened. As he expressed a desire to avoid aggressive intervention, he died peacefully on hospital day 13.

## Discussion

In some developed countries, aging societies have resulted in a large proportion of elderly patients undergoing emergency transportation [6]. Although end-of-life discussions should ideally be held in advance, this is not always the case for all patients [7]. When patients are unable to communicate during emergency transport, treatment is generally provided under the assumption that it is desired. However, the approach to intensive care in elderly patients remains a subject of ongoing debate [8]. Some reports suggest that intensive care should not be withheld based solely on advanced age [9]. Nevertheless, such studies rarely include patients over 95 years of age, as in our case, where ethical considerations must guide clinical decisions. In this case, after the patient’s consciousness improved with asymmetric NHF therapy, it was confirmed that he did not wish to undergo intubation or aggressive treatment. NHF therapy is increasingly used for palliative care because of its comfort, and is also used in home care settings [10,11]. This allows patients to continue oral intake during treatment, as was observed in this case, where oral feeding was possible from the day after admission. This represents a significant advantage over intubation or NIV.

CO₂ narcosis is a form of consciousness disturbance caused by type II respiratory failure, and its treatment generally involves invasive mechanical ventilation or NIV [5]. While intubation provides assured ventilation, it can be difficult to wean elderly patients from mechanical ventilation [12], and patients may refuse such invasive procedures. However, NIV may be ineffective in patients with excessive sputum production or coma [13]. Asymmetric NHF therapy uses an asymmetric nasal cannula to provide high-flow oxygenation and is expected to improve hypercapnia more effectively than conventional NHF by enhancing anatomical dead space washout and providing positive end-expiratory pressure (PEEP) effects [3]. A study comparing asymmetric NHF and conventional NHF in patients with chronic obstructive pulmonary disease (COPD) found a significant reduction in PaCO₂ in patients with baseline PaCO₂ ≥ 65 mmHg in the asymmetric NHF group [14]. In our case, PaCO₂ was elevated at 74 mmHg, a condition in which the effect of asymmetric NHF on lowering PaCO₂ may be more prominent. In hypoxemia without immunodeficiency, cardiogenic pulmonary edema, acute exacerbation of COPD with respiratory acidosis, and COVID-19 pneumonia, NHF has been shown to be non-inferior to NIV in terms of tracheal intubation rate and mortality rate [15]. This suggests that NHF is expected to be as effective as NIV. Patients with impaired consciousness were not included in previous studies on asymmetric NHF therapy, and its efficacy for CO₂ narcosis remains unclear. In this case, pneumonia caused sputum retention and CO₂ narcosis with impaired consciousness, making NIV risky due to the potential for airway obstruction. Therefore, asymmetric NHF therapy was chosen to successfully improve the patient’s CO₂ narcosis. Subsequently, the patient regained the ability to communicate, allowing shared decision-making regarding the treatment plan. This suggests that asymmetric NHF therapy may help to avoid unnecessary intubation and mechanical ventilation.

A PubMed search using the terms "asymmetric nasal high flow" and "narcosis", "asymmetric nasal high flow" and "hypercapnic encephalopathy", as well as "asymmetric nasal high flow" and "hypercapnic coma", yielded no relevant studies. Related literature exists on NHF therapy for preventing CO₂ narcosis by improving hypoventilation during sleep [16]; however, there are no prior reports on the use of NHF specifically for treating CO₂ narcosis. The effect of NHF on ventilation is considered supportive rather than definitive [17], and positive pressure ventilation, including NIV, should not be withheld based on the clinical course alone; however, close monitoring with arterial blood gas analysis is essential. In this case, asymmetric NHF therapy was effective in rapidly lowering PaCO₂ in pneumonia-induced CO₂ narcosis, suggesting its potential utility as a noninvasive respiratory support option in elderly patients.

## Conclusions

Respiratory therapy for elderly patients with type II respiratory failure should be selected based on the patient’s wishes. Further studies and evidence are required to validate the use of asymmetric NHF therapy not only for improving oxygenation but also for managing hypercapnia. In this case, asymmetric NHF therapy was effective in treating CO₂ narcosis triggered by pneumonia in an elderly patient.
